# High-dose vitamin D in a patient with docetaxel-induced photo-recall phenomenon

**DOI:** 10.1016/j.jdcr.2025.02.020

**Published:** 2025-03-11

**Authors:** Sukul Mittal, Joshua Prenner, Jennifer N. Choi

**Affiliations:** Department of Dermatology, Northwestern University Feinberg School of Medicine, Chicago, Illinois

**Keywords:** docetaxel, photo-recall, ultraviolet recall, vitamin D

## Introduction

Photo-recall phenomenon, also known as radiation recall or UV recall, refers to the development of a cutaneous eruption within areas of previous radiation or sunburn following the administration of a systemic drug.^1^ This phenomenon has been primarily reported with methotrexate and antimicrobials but has more rarely been described with various chemotherapeutic agents, including gemcitabine and paclitaxel.^1^^,^^2^ The underlying pathophysiology of photo-recall reactions remains unclear but is hypothesized to involve the reactivation of UV-induced inflammatory pathways upon exposure to the triggering agent.^3^ This phenomenon can be particularly challenging to manage, as it may be quite symptomatic and complicate ongoing chemotherapy regimens.

Docetaxel is a taxane-based microtubule inhibitor that has been implicated in rare cases of photo-recall reactions.^4^^,^^5^ These reactions manifest as erythematous, painful eruptions confined to previously sun-exposed areas and can vary in severity from mild pink erythema to severe blistering with necrosis. In this report, we present a unique case of docetaxel-induced photo-recall in a patient undergoing treatment for breast cancer. In our patient, high-dose vitamin D3 was utilized with the goal of preventing recurrent flares from subsequent exposures, drawing on its studied effectiveness in managing toxic erythema of chemotherapy (TEC).^6^

## Case report

**Fig 1:**
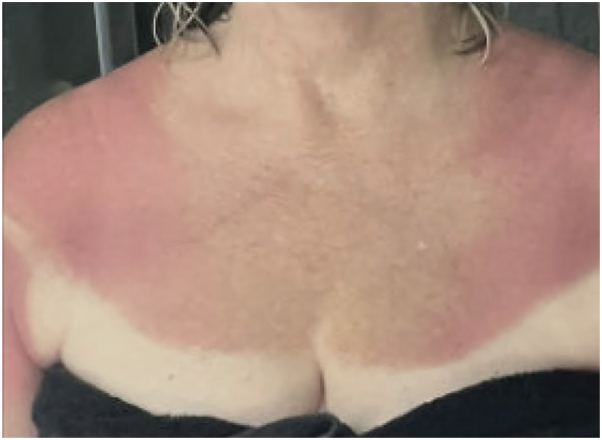
Initial appearance of bright red erythema on sun-exposed areas 2 days after docetaxel infusion.

A 50-year-old female with a history of acute myeloid leukemia in remission and invasive ductal carcinoma of the right breast presented to our clinic with a tender, improving eruption on previously sun-exposed areas of skin. She was receiving treatment for her breast cancer with cyclophosphamide and docetaxel and tolerated her first infusion 4 weeks prior without any cutaneous adverse effects. Three days before her second infusion, she reported spending time outdoors in the sun with her face, upper arms, chest, back, and thighs exposed. She developed what she believed to be a mild, nonblistering sunburn, which turned brown and began to desquamate before her second infusion. However, 2 days after receiving her second infusion of docetaxel, she developed brightly erythematous, tender, and edematous plaques sharply demarcated over the same sun-exposed areas as her recently healed sunburn (Fig 1).

**Fig 2:**
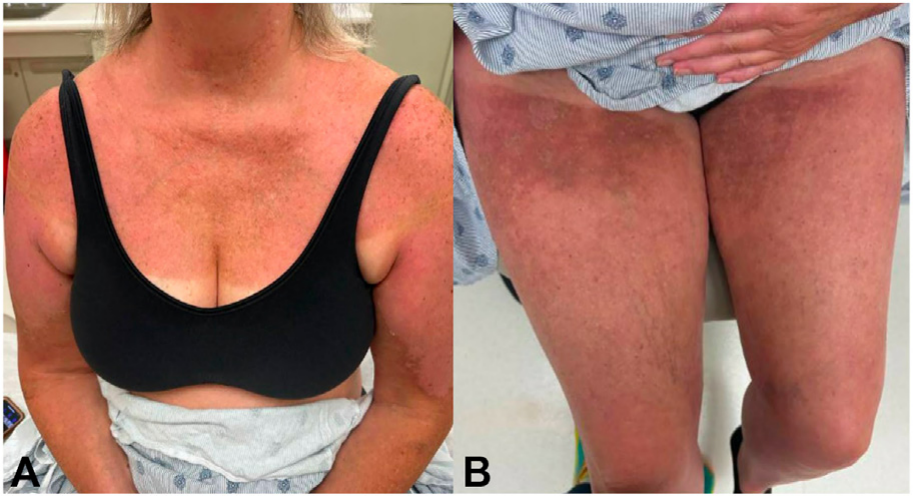
**A,** The chest, arms and **B,** anterior thighs demonstrate ongoing improvement 2 weeks after initial photo-recall reaction without any topical or systemic treatment.

She presented to our dermatology clinic 2 weeks later with interval improvement in the eruption, demonstrating pink to hyperpigmented thin plaques with superficial desquamation on the bilateral shoulders, arms, upper back and chest, and upper thighs (Fig 2). Areas not involved by the initial sunburn were spared. The patient denied using any new topical or oral medications or other exposures since beginning her infusions.

Because the eruption was resolving at the time of presentation, treatment with topical steroids was deferred. However, given the possibility that the photo-recall would recur with future infusions of docetaxel, high-dose oral vitamin D3 (100,000 international units) was prescribed to be taken on the morning of the next scheduled infusion 4 days later.^6^^,^^7^ This dose was based on the study by Nguyen et al, which demonstrated the successful use of 100,000 international units of vitamin D3 in managing TEC.^6^ Following the patient’s third infusion, photo-recall did not occur. She repeated a second dose of 100,000 units of vitamin D3 prior to her fourth and final infusion of docetaxel 3 weeks later and again did not experience recurrence of her eruption. The risk of side effects was considered minimal given that single doses of high-dose vitamin D (up to 600,000 IU) have been shown to reduce inflammation without significantly altering serum calcium or vitamin D levels, and studies in critically ill and oncology patients indicate its use is generally safe without increasing the risk of adverse events.^6^^,^^8^

## Discussion

Photo-recall phenomenon is an adverse cutaneous reaction traditionally associated with methotrexate. Herein, we describe a case of photo-recall reaction to docetaxel, a chemotherapeutic agent commonly used to treat a variety of malignancies. While the pathophysiology of these reactions is not fully understood, previously sensitized memory T cells are likely implicated in the reactivation of UV-induced inflammatory pathways.^3^

Our patient may have benefitted from the administration of high-dose oral vitamin D3 prior to her subsequent infusions of docetaxel. This recommendation was based on the documented efficacy of high-dose vitamin D3 in the treatment of both UV-induced solar erythema and TEC in hospitalized patients.^6^^,^^7^ Vitamin D3 is known to exhibit various immunomodulatory and anti-inflammatory properties, which may explain its potential success in preventing and reducing cutaneous inflammation in photo-recall and similar reactions.^7^ One proposed mechanism is that vitamin D3 promotes the upregulation of arginase-1, leading to the induction of M2-polarized, anti-inflammatory macrophages, which aid in resolving inflammation.^7^ Additionally, vitamin D3 has been shown to reduce DNA damage and keratinocyte apoptosis following UV exposure, which may contribute to its protective effects in phototoxic conditions.^7^ These mechanisms together suggest that vitamin D3 may play a role in reducing the inflammation and tissue damage associated with docetaxel-induced photo-recall.

In one reported case of docetaxel-induced photo-recall, docetaxel had to be discontinued due to the severity of the reaction.^5^ Another report of a patient with docetaxel-induced photo-recall required switching to paclitaxel, an alternative taxane-based agent.^4^ It is important to note that to date, no documented cases have shown recurrence of photo-recall after continuing taxane-based therapy, as patients either discontinued treatment or switched to an alternative due to the severity of their photo-recall reaction. However, while recurrence of photo-recall reactions has not been reported with taxane-based therapies, other cutaneous reactions such as immediate hypersensitivity reactions, macular and papular eruptions, and folliculitis have been shown to recur with continued therapy.^9^ Furthermore, another study found that drug rechallenge in cases of radiation-recall dermatitis triggered by cytotoxic drugs could cause mild to severe flares.^10^ Although we cannot know if our patient would have experienced a recurrence, it is notable that she was able to successfully complete her treatment without issue. To our knowledge, this is the first case to explore the use of high-dose vitamin D3 in a case of photo-recall after docetaxel. While future studies are needed to further explore the role of vitamin D3 in treating photo-recall reactions, our case suggests that vitamin D3 may have a possible role to play in the care of these patients.

## Conflicts of interest

None disclosed.
